# A Panel of DNA Methylation and Proteomic Biomarkers for Specific Aging Pathways

**DOI:** 10.1093/geroni/igaa057.423

**Published:** 2020-12-16

**Authors:** Albert Higgins-Chen, Luigi Ferrucci, Morgan Levine

**Affiliations:** 1 Yale School of Medicine, New Haven, Connecticut, United States; 2 National Institute on Aging, Bethesda, Maryland, United States; 3 Yale University School of Medicine, New Haven, Connecticut, United States

## Abstract

Most aging biomarkers such as DNA methylation and proteomic clocks have focused on measuring overall “biological age,” a single number that predicts age-related morbidity and mortality better than absolute chronological age. While intuitive and interpretable, this single biological age number does not account for the possibility that different individuals may preferentially experience aging in different molecular and cellular pathways, and therefore does not suggest personalized aging interventions. We reasoned that a panel of biomarkers each capturing specific aging pathways, such as mitochondrial dysfunction or cellular senescence, may capture the heterogeneity of aging better than existing composite measures. To address this, we employed weighted gene co-expression network analysis to cluster tissue-specific transcriptomes and the serum proteome into specific modules with distinct biological functions and characterized how these modules change with age. We trained DNA methylation proxies of these functional modules that we then applied to independent validation data to identify associations with age-related morbidity and mortality. Clustering analysis using the DNA methylation biomarkers showed that different individuals show distinct patterns of aging. These pathway-specific biomarkers will elucidate how different aging mechanisms interact with each other to produce the larger phenomenon of aging, and for evaluating novel therapeutics targeting specific hallmarks of aging.

